# Incidence Dependency of Photonic Crystal Substrate and Its Application on Solar Energy Conversion: Ag_2_S Sensitized WO_3_ in FTO Photonic Crystal Film

**DOI:** 10.3390/ma12162558

**Published:** 2019-08-11

**Authors:** Xi Ke, Mengmeng Yang, Weizhe Wang, Dongxiang Luo, Menglong Zhang

**Affiliations:** 1 Institute of Semiconductors, South China Normal University, Guangzhou 510631, China; 2 School of Materials and Energy, Guangdong University of Technology, Guangzhou 510006, China

**Keywords:** photocatalytic materials, photonic crystals, nanocomposites

## Abstract

In addition to the most common applications of macroporous film: Supplying a large surface area, PC-FTO (macroporous fluorine-doped tin oxide with photonic crystal structure) can be employed as a template to control the morphologies of WO_3_ for exposing a more active facet, and enhance the overall photo-electron conversion efficiency for the embedded photoactive materials under changing illumination incidence through refracting and scattering. The optical features of PC-FTO film was demonstrated by DRUVS (diffuse reflectance UV-vis spectra). Plate-like WO_3_ were directly synthesized inside the PC-FTO film as a control group photoanode, Ag_2_S quantum dots were subsequently decorated on WO_3_ to tune the light absorption range. The impact of photonic crystal film on the photoactivity of Ag_2_S/WO_3_ was demonstrated by using the photoelectrochemical current density as a function of the incidence of the simulated light source.

## 1. Introduction

Photocatalysis for applications on a photon-electricity or photon-chemical bond have been paid much attention due to the energy crisis and environmental pollution [1]. Photoelectrochemical devices were therefore developed to perform solar energy conversion including photocurrent generation [2], pollutant degradation [3], and H_2_ or CO evolution [4,5]. In consideration of the photocatalysis mechanism, improving the performance of a solar energy conversion device is based on several critical factors, such as the enhancement of light harvesting, efficient utilization of photons, rapid charge carrier separation and transportation. To this end, many efforts including elemental doping [6], dye sensitization [7], adding co-catalysts [8], making composites [9], plasma enhancement [10] and nanostructuring the photoactive materials [11,12] have been made, leading to improved solar energy conversion efficiency being achieved. Though, in addition to the above attempts, a more convenient strategy is correlating the nature of the sun and materials. To be more specific, the sun can be considered as a light source of changing angle relative to a certain location of the Earth, due to the changing solar elevation angle. Thus, the solar energy conversion devices receive photons of varied incidence and density, typically, the photon density (mW cm^−2^) reaches its zenith at noon (ca. 90° solar elevation angle). In this case, the photonic crystals (PCs) could take advantage of this nature of the sun for more effective utilization of solar energy, due to its capability for manipulating the photon migration depending on their incidence.

PCs are long-range ordered arrays materials and possess periodic modulation of refractive index (RI) within an optical wavelength. The formation of photonic stop band (PSB) of a certain PCs structural material is attributed to the differences between the RI of the PCs material and the RI of its external media (including gas, liquid or solid), and the periodicity. The concept of photonic effect within the PSB range in PCs was first proposed by John [13] and Yablonovitch [14], independently. After that, the photonic properties of PCs were further explored by theoretical calculations [15,16,17]. That is, within a particular wavelength range that satisfies Bragg-Snell’s law [18], the PSB inhibits the propagation of photons from a certain direction, reduces the group speed of photons (slow photons) and induces multiple scattering [19]. The position of PSB in wavelength is commonly tunable, as is demonstrated by Bragg-Snell’s law, it is reliant on the periodic order, the RI of PCs material and external media, filling factors and incidence of illumination. Whereas for a given PCs based solar energy conversion device in certain environment, filling factor, periodic order and RI should be considered as constants, and thus the incidence of illumination would be the only tunable parameter for the manipulation of PSB positions. In addition, different from tuning the RI, the filling factor or periodic order, the changing of incidence would not affect the other properties of PCs (including geometric surface area, electric conductivity and interfaces between PCs and embedded photoactive materials), which makes it very convenient to study the PSB impact on photoactivity of embedded photoactive materials.

TCOs (transparent conductive oxides) including ITO (indium-doped tin oxide), ATO (antimony-doped tin oxide) and FTO (fluorine-doped tin oxide) have been intensively applied in functional windows, solar cells and displays [2,20]. For a solar energy conversion device, TCOs are an ideal material for the synthesis of PCs due to their transparency in visible range and good electrical conductivity. For instance, in previous studies, powder or film PCs have been employed to supply enlarged surface areas available to support the embedded photoactive materials including g-C_3_N_4_ [21], CdS [22], TiO_2_ [23], and halide perovskite photocatalyst [24]. Although the studies of the photonic effect of PSB towards photoactivity of embedded photoactive material are limited. Of direct relevance to this work, CdS embedded WO_3_ PCs powders were fabricated [25], the manipulation of PSB was obtained through varying the periodic order (pore size) of WO_3_ PCs powder. Enhancement of the hydrogen evolution rate is achieved in case of absorption range of CdS overlapping with the PSB of WO_3_ PCs. However, the varied periodic order of WO_3_ PCs would not only change the PSB but also suggest a different surface area and reaction sites for the embedded photoactive materials, which make it less convenient to study the photonic effect on photocatalysis. In our recent report [26], g-C_3_N_4_ as the photoactive material was embedded in an FTO PCs film, overlapped the PSB of FTO PCs film and absorption range of g-C_3_N_4_ was achieved by tuning the position of the PSB through controlling incidences. An alternative approach to fit the PSB and absorption range of photoactive materials is tuning the absorption of photoactive materials by adding dyes.

Inorganic semiconductors such as CdS, CdSe, PbS, MnTe, and Ag_2_S, or organic dyes have been exploited as sensitizers for light-absorption enhancement in dye-sensitized solar cells (DSSCs) or quantum dot-sensitized solar cells (QDSCs) [3,9]. Among them, Ag_2_S is a convenient candidate compound for applications in photoanodes. The energy band gap of Ag_2_S is ca. 1.1 eV [27], corresponding to a broad absorption range of visible light and near-IR regions. In addition, Ag_2_S reveals a promising absorption coefficient of ca. 104 cm^−1^ [28]. In the previous study, Ag_2_S quantum dots were prepared on TiO_2_ of various architectures (including nanotubes and nanorods) [29] for the formation of DSSCs, and also being synthesized on ZnO and SnO_2_ for the preparation of QDSCs [30,31]. The adding of Ag_2_S exhibited impressive light absorbability for the electrodes and thus led to improved photoactivity. However, in addition to these wide bandgap semiconductors (TiO_2_, ZnO and SnO_2_), WO_3_ exhibits a narrower band gap which can be photoactivated by visible light, and the band structure allows an efficient photoelectron injection when composited with Ag_2_S [32].

In this work, WO_3_ was exploited as the photoactive materials, plate-like WO_3_ were directly synthesized in FTO PCs film via a solvothermal method. Ag_2_S was then loaded on WO_3_ via SILAR (successive ion layer adsorption and reaction) method. As control groups, WO_3_ and Ag_2_S sensitized WO_3_ were also prepared on p-FTO (planar FTO glass). DRUVS (diffuse reflectance UV-vis spectra) and PEC (photoelectrochemistry) were correlated to analyze the photonic effect on the photocatalytic performance of Ag_2_S sensitized WO_3_.

## 2. Materials and Methods

H_2_SO_4_ (≥95%), H_2_O_2_ (30 vol.%), FTO glass slide (11 Ω sq^−1^), monodispersed polystyrene (2.5 wt.% aqueous suspension, 450 nm in diameter), SnCl_4_·5H_2_O (99.99%), NH_4_F (99.99%), Na_2_S·9H_2_O (99.5%), AgNO_3_ (99.8%), WCl_6_ (99.95%) and methanol were purchased from Sigma-Aldrich (Saint Louis, MO, USA) and used as received.

### 2.1. Synthesis of PC-FTO Films

The PC-FTO film was prepared through a modified well-established soft-template method [33]. A planar FTO glass was stood vertically in a glass sample vial (10 mL volume) containing 5 mL of PS aqueous suspension at 60 °C for 16 h to obtain the PS film template. The FTO precursor solution was prepared from SnCl_4_·5H_2_O (1.4 g, 4 mmol) sonicated in ethanol (20 mL) until dissolved, next, the saturated NH_4_F solution (0.24 g, 2 mmol) was added, and the resulting mixture was further sonicated until optically clear and colorless. The PS film was pre-soaked in ethanol for 30 min before being stood vertically and submerged in FTO precursor solution for another 30 min under a vacuum (0.1 Pa). The wet slide was then removed from the glass vial and transferred to a furnace oven for calcination at 450 °C for 2 h with a heat ramp rate of 1 °C min^−1^ in the air to burn off the PS spheres.

### 2.2. In-Situ Synthesis of WO_3_ Platelets in PC-FTO Films

The WO_3_ precursor solution was prepared using WCl_6_ (1.0 g, 6 mmol) sonicated in methanol for ca. 15 min (40 mL) until dissolved. A PC-FTO substrate was put in an autoclave (25 mL volume) filled with 15 mL WO_3_ precursor solution. Subsequently, the autoclave was transferred to a furnace oven at 100 °C for 6 h. The sample was then transferred from the autoclave to a furnace oven for a heat treatment at 475 °C for 2 h with a heat ramp rate of 1 °C min^−1^ in the air.

### 2.3. Sensitizing of WO_3_ with Ag_2_S Quantum Dots via SILAR Method

The Ag_2_S precursor was prepared from AgNO_3_ (50 mM) and Na_2_S (50 mM) aqueous solutions. Typically, a WO_3_@PC-FTO film was soaked in AgNO_3_ solution for 30 s before being dried by nitrogen stream. The film was then soaked in Na_2_S solution for another 30 s. Subsequently, the film was rinsed with DI water and dried with nitrogen stream. This process was repeated several times to get Ag_2_S/WO_3_@mac-FTO photoanodes sensitized with the varied amounts of Ag_2_S dots.

### 2.4. Characterization

Samples were stuck to an aluminium stage by sticky carbon tape, and then the SEM images were achieved by a Hitachi S-4800 field emission scanning electron microscope (Tokyo, Japan). For the preparation of TEM samples, films were scraped off from the FTO substrates and carefully ground. The samples were then transferred to methanol for 15 min sonication. One drop of the suspension was added to 3 mm porous carbon-coated copper grids and allowed to dry under air. JEOL 2011 transmission electron microscopes (Tokyo, Japan) were exploited to collect the TEM images with 200 kV accelerating voltage. An attached kit of an EDAX Phoenix X-ray spectrometer (Mahwah, NJ, USA) incorporated to the TEM was employed to perform energy dispersive analysis of X-rays (EDX) mapping. An Ocean Optics HR2000+ high-resolution spectrometer (Edinburgh, UK) was incorporated with a DH-2000-BAL lamp with a light wavelength of 200 nm to 1100 nm (deuterium/helium). An R400-7-UV-Vis transmission probe (Ocean Optics, Edinburgh, UK) was used to record the diffuse reflectance UV-vis spectra using deuterium/helium lamb (200 nm–1100 nm). Spectra were collected using Spectra Suite software (Version 2.7) of 10 s integration time, 30 boxcar smoothing width, and 10 scans to average. Wide angle PXRD patterns were obtained by a Lynx eye incorporated detector Bruker-AXS D8 Advance instrument (Billerica, MA, USA), with Cu Kα (1.54Å) radiation, the slit on the source was 1 mm and the detector slit was 2.5 mm. PXRD data were scanned from 10° to 70° 2θ, with 0.02° step size and a scan speed of 0.1 s per step.

PEC measurements were carried out using a standard 3 electrode setup. The Ag/AgCl (3 M KCl internal solution) was exploited as the reference electrode and a platinum sheet (10 × 10 mm^2^) was used as the counter electrode. The samples were used as a working electrode, the connection was achieved using copper tape on the top of the electrode and the bottom 10 mm of the electrode was immersed into the electrolyte solution. The PEC cell contained a quartz window allowing the illumination of simulated solar light. A 150 W Xe lamp with the (irradiance of ca. 100 mW cm^−2^) was exploited as the simulated light source. NaOH (1 M) with pH at 13.6 was prepared as the aqueous electrolyte solution using the Millipore system (≥18 MΩ cm^−3^, Burlington, MA, USA) filter water. All potentials can be referenced to the reversible hydrogen electrode (RHE) using the following equation: E_ref_(Ag/AgCl) = 0.0210 V vs. NHE at 25 °C.

$$E\left(\mathrm{vs}.\;\mathrm{RHE}\right)\;=\;E\left(\mathrm{vs}.\;\mathrm{Ag}/\mathrm{AgCl}\right)+E_{\mathrm{ref}}\left(\mathrm{Ag}/\mathrm{AgCl}\right)+0.0591V\times \mathrm{pH}$$

## 3. Result and Discussion

### 3.1. Geometrical Properties

**Figure 1:**
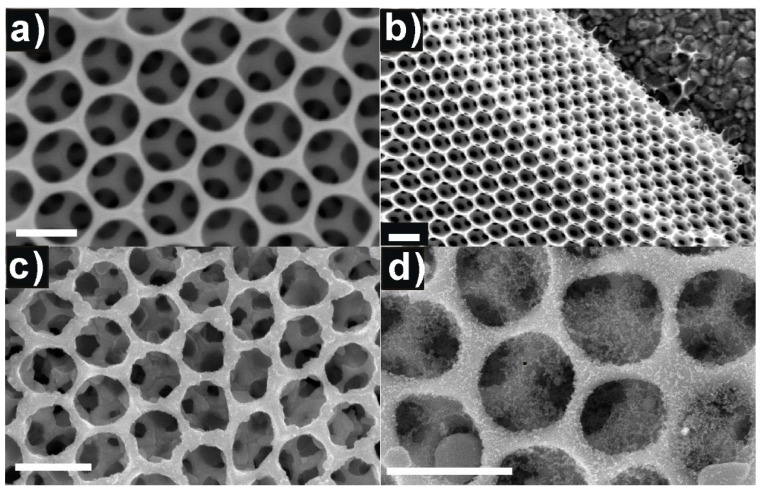
(**a**) SEM image of macroporous fluorine-doped tin oxide with photonic crystal structure (PC-FTO) viewed perpendicularly; (**b**) SEM image of the edge of PC-FTO film; (**c**) SEM image of WO_3_@PC-FTO; (**d**) SEM image of Ag_2_S/WO_3_@PC-FTO, scale bar = 500 nm.

The geometrical properties of the as-prepared PC-FTO films are initially characterized by scanning electron microscope (SEM), Figure 1a exhibits an inverse opal structural (face-centered cubic, FCC) film with the pore size of ca. 330 ± 30 nm and a smooth FTO skeleton. This PC-FTO film was fabricated on FTO glass via a well-established soft template method [34]. Figure 1b presents the edge of the as-prepared PC-FTO film, which was approximately nine layers corresponding to ca. 2 µm in thickness, indicating a large surface area available for the embedded materials, in comparison to that of planar analogs. The long-range ordered FCC array and the periodic RI (between FTO skeleton and air pore) suggests intensive photonic effects as expected by Bragg-Snell’s law. The hexagonal arrangement of the air pore corresponds to the (111) plane of an FCC structure, which is the predominant plane for the formation of a PSB from a PCs film. After the coating of WO_3_, homogeneous plate-like materials could be observed on the skeleton of PC-FTO (Figure 1c and Figure S1), without blocking the pores. The WO_3_@PC-FTO exhibits a relatively rougher surface in comparison to the bare PC-FTO (Figure 1a).

**Figure 2:**
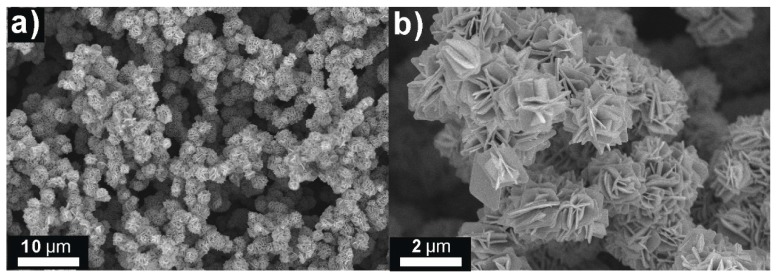
SEM images of WO_3_ synthesized on planar FTO glass. (**a**) SEM of WO_3_ of low magnification; (**b**) SEM of WO_3_ of high magnification.

It should be noted that the amount of the embedded photoactive materials in a PC film should be carefully controlled because the excessive coating will reduce the periodicity of PCs, on the other hand, the insufficient coating may lead to weak photoresponse. Furthermore, the geometrical shape of the WO_3_ in a PC-FTO film can also be compared to that which is synthesized on a planar FTO substrate using the same method (Figure 2). Within PC-FTO films, the feature size of the materials can be restrained to a certain scale by the nanostructural substrate due to the complex geometrical surface, similar feature size control effects can also be found in Fe_2_O_3_ embedded SiO_2_ [35] or SnO_2_ [12] in previous reports. Figure 1d and Figure S2 reveal the WO_3_@PC-FTO film after the decoration of Ag_2_S, the Ag_2_S decorated samples led to rougher surface due to the relatively small crystal size of Ag_2_S.

### 3.2. Optical Properties

**Figure 3:**
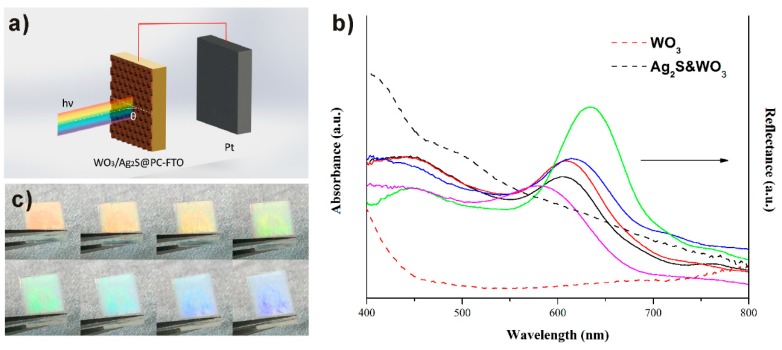
(**a**) Schematic diagram of the varied incidence illumination on a PC-FTO based electrode. (**b**) diffuse reflectance UV-vis spectra (DRUVS) (solid line) of photonic stop band (PSB) positions under different incidence and UV-vis absorption spectra (dash line) of WO_3_ and Ag_2_S/WO3. (**c**) digital photographs of one PC-FTO film under different incidences.

Since this work was focused on the impact of photonic crystal film on the photocatalytic performance of Ag_2_S/WO_3_, the photonic effects and the optical properties of Ag_2_S/WO_3_ were characterized by diffuse reflectance UV-vis spectra (DRUVS) and UV-vis absorption spectra, respectively. The PSB position (expressed as the reflectance maximum) of PC-FTO film was recorded at the different incidence of illumination with respect to the surface normal (Figure 3a) through DRUVS. For comparison, the PSB position for (hkl) plane of a PC-FTO was also estimated by mathematic calculation from a combination of Bragg’s and Snell’s laws as the following expresses:

λ =2dhklm[φn2+(1−φ)n02−sin2θ]12

dhkl= D2[h2+k2+l2]12

where *φ* is the filling factor of PC-FTO film (assumed to be 0.26 for FCC structure), *n* and *n*_0_ are the RI of FTO (ca. 1.61) and the external media, respectively. D is the periodic order of PC-FTO film (ca. 340 nm). Since the calculation of a PSB position using Bragg-Snell’s law is based on an ideal FCC structural model, thus a comparison between the calculated and experimental incident dependence of PSB can be exploited to evaluate the optical quality of the as-prepared PC-FTO film. As shown in Table S1, the experimental PSB is in good agreement with the Bragg diffraction from (111) sets of planes, in an incidence range from 15° to 45°. The PC-FTO exhibits varied PSB positions (from 585 nm to 635 nm) in the DRUVS (Figure 3b) according to the incidence of illumination. The PSB shift can also be confirmed by eye when holding the PC-FTO film at a certain angle relative to the lamp, the colors (attributed to the diffuse reflectance of PCs) varied from red to violet as shown in Figure 3c, indicating a wide tunable range of PSB positions. It should be noted that the DRUVS can hardly collect the reflected photons when there is a wide angle between sample and probe, but the photographs in Figure 3c could be evidence for the wide tunable range of the PSB.

### 3.3. Qualitative Analysis

**Figure 4:**
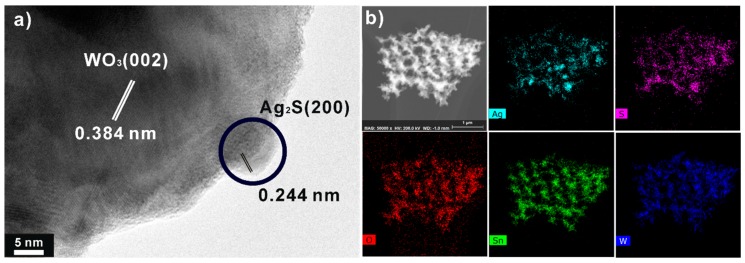
(**a**) high-resolution transmission electron microscopy (HR-TEM) image of Ag_2_S/WO_3_@PC-FTO photoanode; (**b**) TEM-EDX elemental mapping of Ag_2_S/WO_3_@PC-FTO.

**Figure 5:**
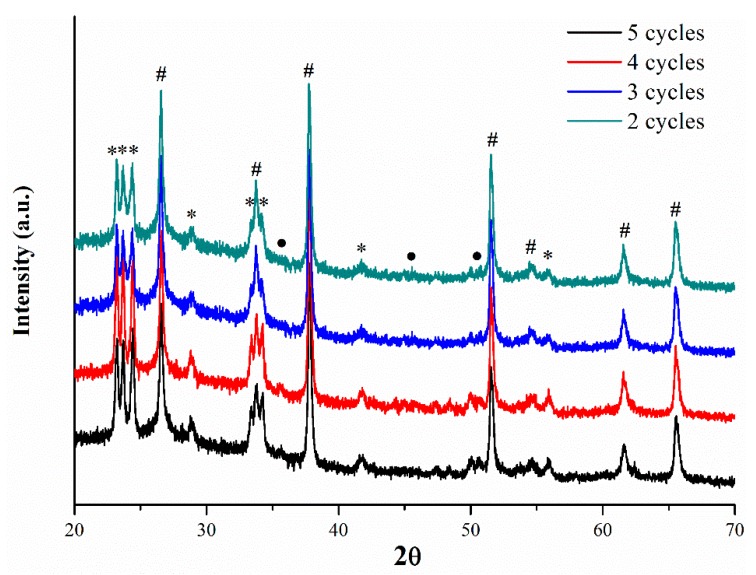
Powder X-ray diffraction (PXRD) of Ag_2_S/WO_3_@PC-FTO electrode with different loading of Ag_2_S, * = WO_3_, # = SnO_2_ and • = Ag_2_S.

Figure 4a and Figure S4 reveal the high-resolution transmission electron microscopy (HR-TEM) images of the as-prepared Ag_2_S/WO_3_ on PC-FTO, the embedded WO_3_ platelets were attached by Ag_2_S (few nanometer in size), in which the lattice fringes corresponded to the (200) plane of Ag_2_S (JCPDS 04-0774) and the (002) crystal plane of WO_3_ (JCPDS 43-1034), respectively. The elemental mapping of Ag_2_S/WO_3_@PC-FTO was achieved by energy dispersive X-ray spectroscopy (EDX), from which the skeleton of PC-FTO, WO_3_ platelets and Ag_2_S dots are clearly presented in Figure 4b and Figure S3. The elemental ratio of the as-prepared Ag_2_S/WO_3_@PC-FTO sample was presented in Table S2. The powder X-ray diffraction (PXRD) was also employed for the characterization of an as-prepared Ag_2_S/WO_3_@PC-FTO, from which the diffraction patterns were consistent with the HR-TEM results. The loading amounts of Ag_2_S via SILAR led to no obvious difference in the PXRD patterns of samples but the slightly increased diffraction patterns from Ag_2_S (Figure 5).

### 3.4. Photocatalytic Properties

Photoelectrochemistry (PEC) was exploited to evaluate the photoactivity of Ag_2_S/WO_3_@PC-FTO photoanode, as a function of incidence. The setup of a PEC cell was built up with a standard three electrode system in 1 M NaOH aqueous electrolyte, in which the sample, Pt wire, and Ag/AgCl were employed as a working electrode, counter electrode and reference electrode, respectively. The incidence of illumination was obtained by rotating the working electrode to a certain degree relative to the Xe lamp (as shown in the schematic diagram in Figure 3a). Since the rotating of electrode will lead to a reduction of received photon density (mW cm^−2^) by electrode, the photo density received by electrode at a certain angle was obtained by rotating a detector (shell removed) of photometer at the same angle, thus a correction (photocurrent divided by photon density received by photometer at certain angle) was conducted to evaluate the photocurrent generation under constant irradiance (1 sun, 100 mW cm^−2^).

Ag_2_S/WO_3_@PC-FTO electrodes of different SILAR cycles performed varied photocatalytic activities when the surface normal was illuminated, in which the 3 SILAR cycles performed the maximum photocurrent density of ca. 1.54 mA cm^−2^ at 0 V vs. Ag/AgCl (Figure S5) and good stability (Figure S7) in a linear sweep voltammogram (LSV), and thus this electrode was selected for the study of incidence. 

**Figure 6:**
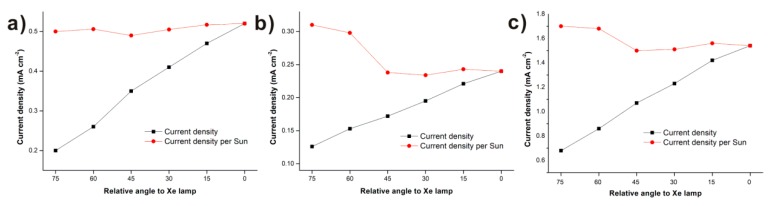
Incidence dependence of photoelectrochemistry (PEC) current density and current density relative to 1 sun (ca. 100 mW cm^−2^) for (**a**) Ag_2_S/WO_3_@planar-FTO; (**b**) WO_3_@PC-FTO and (**c**) Ag_2_S/WO_3_@PC-FTO at 0 V vis V_Ag/AgCl_, the irradiance to the surface normal is adjusted to 1 sun.

**Scheme 1:**
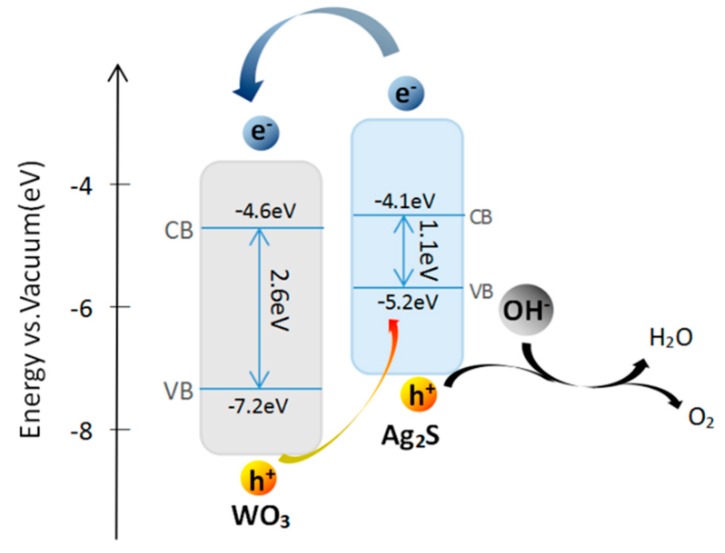
Mechanism of Ag_2_S decorated WO_3_ under illumination.

For comparison, the Ag_2_S/WO_3_@planar-FTO photoanode and WO_3_@PC-FTO photoanode were also prepared using the same method. Due to the absence of PCs substrate, the current density generated by Ag_2_S/WO_3_@planar-FTO is ca. 32% (0.52 mA cm^−2^ vs. 1.54 mA cm^−2^) in comparison to Ag_2_S/WO_3_@PC-FTO at surface normal illumination, this is due to the lack of surface area available for the photoactive materials. The current density of Ag_2_S/WO_3_@planar-FTO was reduced 61.5% along with the incidence from 0° to 75° (Figure 6a), while the current density per sun shows no obvious variation, suggesting that the electrode could generate stable current density relative to 1 sun. In terms of the WO_3_@PC-FTO electrode, the current density reduced 47.6% from 0° to 75° illumination (Figure 6b). Interestingly, an obvious enhanced current density per sun was obtained at 75° and 60° illumination, where the edge of the PSB (60°) and PSB (75°) overlapped with the absorption range of WO_3_. Though for the Ag_2_S/WO_3_@PC-FTO electrode (Figure 6c), due the narrow band gap of Ag_2_S (1.1 eV, Scheme 1) [36] in visible, the PSB of PC-FTO can always overlap with the absorption range of Ag_2_S/WO_3_, and thus reducing the enhancement of current density per sun when rotating the electrode, but it still performed a slower reduction (54.5%) of current density with respect to that on planar-FTO. In theory, the PCs could suppress the recombination of charge carriers in PSB or its edge [37], and this was proved in some previous experimental reports, for instance, a 40% longer lifetime of PL decay was observed when the emission frequency of Tb^3+^ is close to the PSB in a PC-SiO_2_ system [38]. With respect for a photocatalysis system, the external media is typically aqueous (RI ≥ 1.33), and thus the RI differences between PCs materials and external media is smaller than PCs in the air (RI ≈ 1.00), which may reduce the photonic effects from PSB (Figure S6). For example, the g-C_3_N_4_ exhibited no excited state lifetime change when its absorption range overlaps with the PSB [8]. In our case, although the absorption of Ag_2_S/WO_3_ always overlapped with the PSB, we also saw no obvious PL decay lifetime enhancement. Even though, the scattering and refracting will still improve the utilization of light, when the incidence of light changes. The observed slower reduction of current density and the improved current density per sun at certain incidence suggests the realistic value of PCs.

## 4. Conclusions

Ag_2_S/WO_3_ and WO_3_ were synthesized in a PC-FTO film to demonstrate the incidence of light as a function of photocurrent density. In addition to the enhanced surface area, the embedded photoactive materials in PC-FTO perform enhanced PEC at certain incidences in comparison to that on planar analogs. Due to the multiple scattering and refracting nature of PCs, photons could be collected more effectively at certain incidence ranges (in comparison to planar analogs), resulting in enhanced photocurrent generation when integrating the product from all incidences. In consideration of the nature of sun (a changing angle light source), the results suggest that the PCs based substrate would support more efficient utilization of solar energy for the embedded photoactive materials.

## Supplementary Materials

The following are available online at https://www.mdpi.com/1996-1944/12/16/2558/s1, Figure S1: SEM image of a bare WO_3_@mac-FTO photoanode; Figure S2: SEM images of mac-FTO film coated with only Ag_2_S quantum dots; Figure S3: EDX elemental mapping of Ag_2_S/WO_3_@mac-FTO with 3 SILAR cycles; Figure S4: TEM of Ag_2_S/WO_3_@PC-FTO in different magnification; Figure S5: (a) UV-vis absorption and (b) LSV of Ag_2_S/WO_3_@PC-FTO with different SILAR cycles; Figure S6: PSB spectra of PC-FTO in NaOH electrolyte; Figure S7: Stability test of Ag_2_S/WO_3_@PC-FTO electrode under illumination from surface normal for 5 min; Table S1: Calculated and experimental PSB of PC-FTO film from (111) plane; Table S2: Elemental ratios of Ag_2_S/WO_3_@PC-FTO with 3 SILAR cycles.
