# Supplementary material for: Incidence Dependency of Photonic Crystal Substrate and Its Application on Solar Energy Conversion: Ag2S Sensitized WO3 in FTO Photonic Crystal Film
Source: Materials (Basel). 2019 Aug 11;12(16):2558. doi: 10.3390/ma12162558 (PMC6720774; doi:10.3390/ma12162558)
Supplement: Supplementary file 1 [file materials-12-02558-s001.pdf]

materials

Supplementary

Article

Incidence Dependency of Photonic Crystal Substrate and Its Application on Solar Energy Conversion: Ag_2S Sensitized WO_3 in FTO Photonic Crystal Film

Xi Ke [1], Mengmeng Yang [2], Weizhe Wang [1], Dongxiang Luo [2,*] and Menglong Zhang [1,*]

[1] Institute of Semiconductors, South China Normal University, Guangzhou 510631, China
[2] School of Materials and Energy, Guangdong University of Technology, Guangzhou 510006, China
* Correspondence: luodx@gdut.edu.cn (D.L.); mlzhang@scnu.edu.cn (M.Z.)

Received: 7 July 2019; Accepted: 8 August 2019; Published: 11 August 2019

Table S1. Calculated and experimental PSB of PC-FTO film from (111) plane.

Incidence (degree)	Calculated PSB (nm)	Experimental PSB (nm)
15	643	635
30	581	607
45	514	585
60	438	-
75	374	-

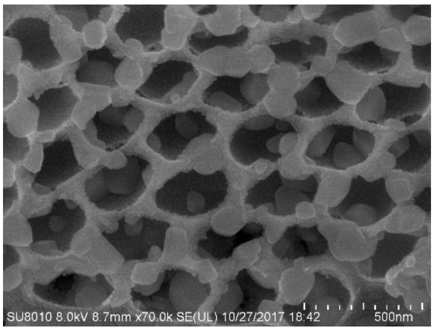

Figure S1. SEM image of bare WO_3@mac-FTO photoanode.

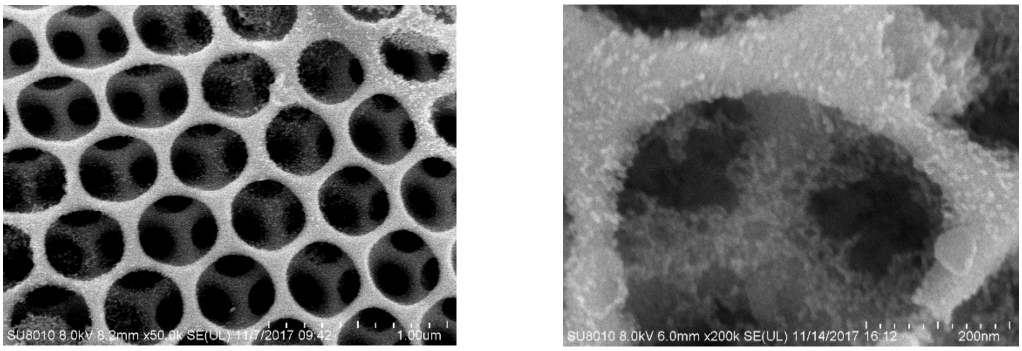

(**a**) (**b**)

Figure S2. SEM images of mac-FTO film coated with only Ag_2S quantum dots.

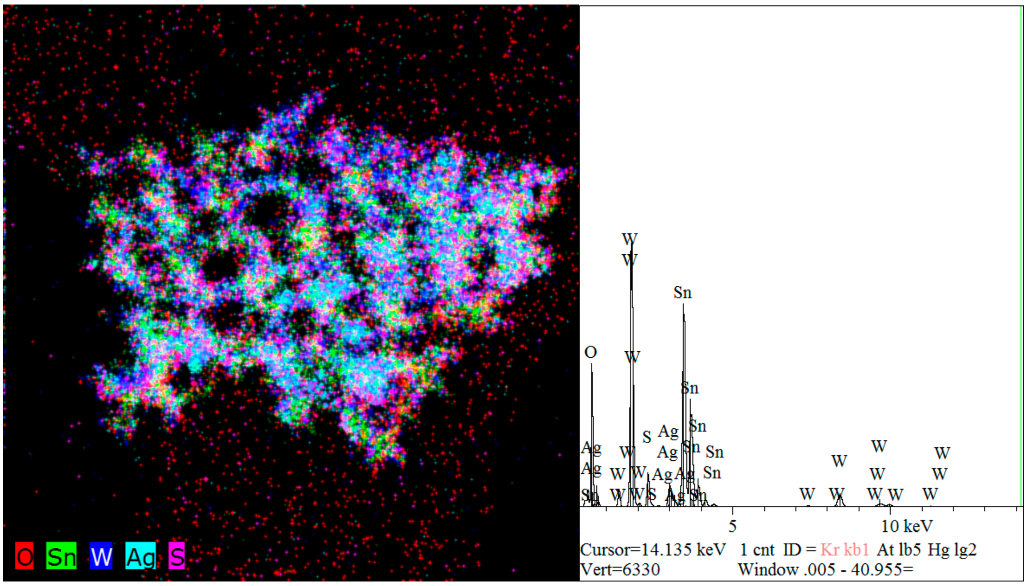

Figure S3. EDX elemental mapping of Ag_2S/WO_3@mac-FTO with 3 SILAR cycles.

Table S2. Elemental ratios of Ag_2S/WO_3@PC-FTO with 3 SILAR cycles.

Elt.	Line	Intensity (c/s)	Atomic %	Atomic Ratio	Conc	Units	Error 2-sig	MDL 3-sig	
O	Ka	105.08	53.736	1.0000	12.344	wt.%	0.257	0.138	
S	Ka	37.46	2.749	0.0512	1.266	wt.%	0.061	0.070	
Ag	La	23.64	2.149	0.0400	3.328	wt.%	0.252	0.324	
Sn	La	276.14	27.941	0.5200	47.625	wt.%	0.635	0.419	
W	La	26.00	13.425	0.2498	35.437	wt.%	2.003	2.207	
			100.000		100.000	wt.%			Total

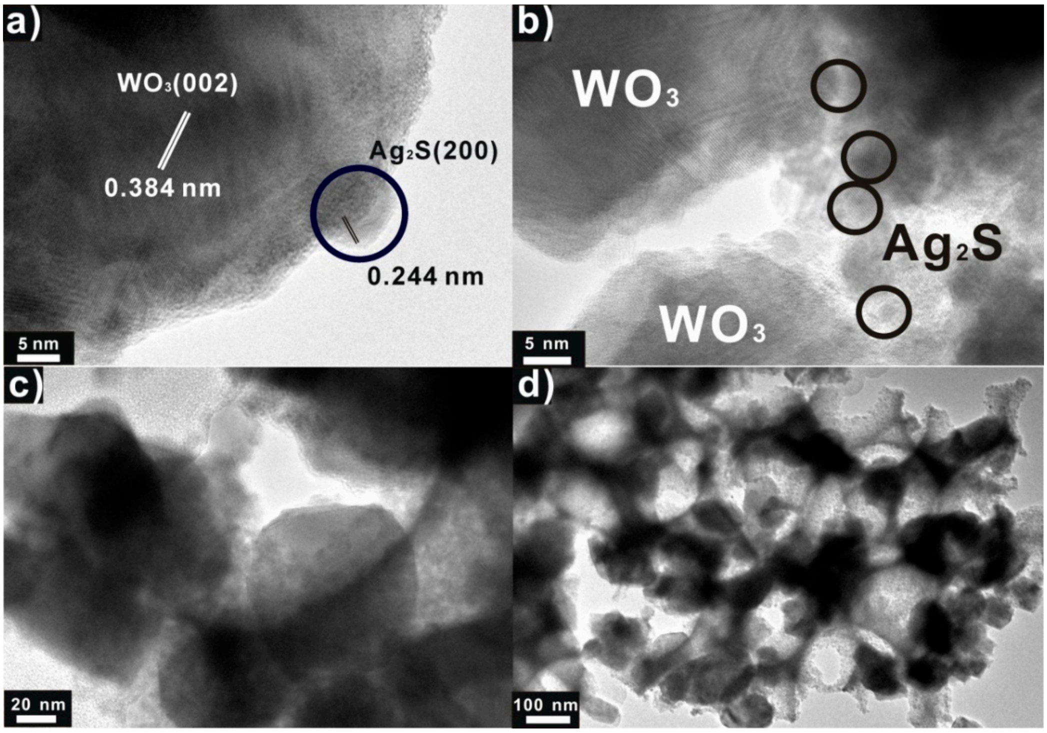

Figure S4. TEM of Ag_2S/WO_3@PC-FTO in different magnification.

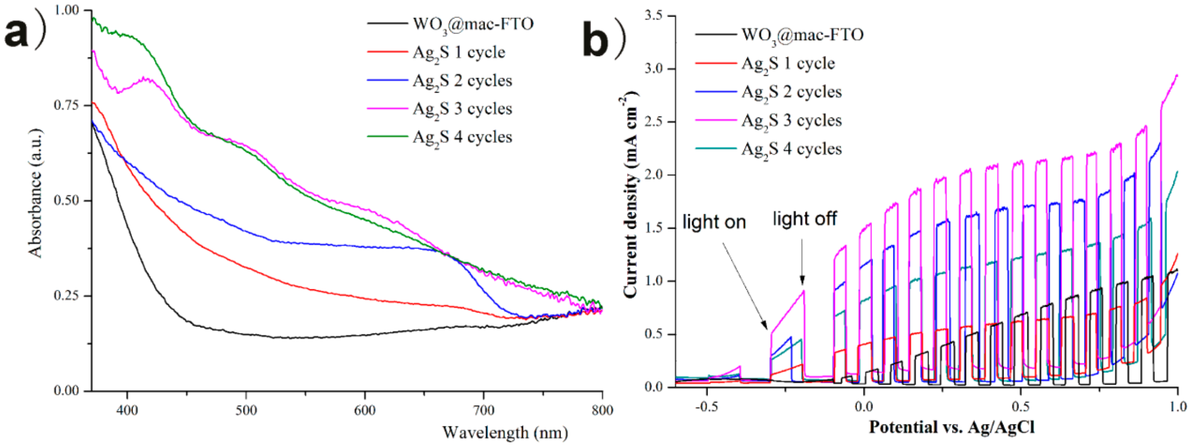

Figure S5. (**a**) UV-vis absorption and (**b**) LSV of Ag2S/WO3@PC-FTO with different SILAR cycles.

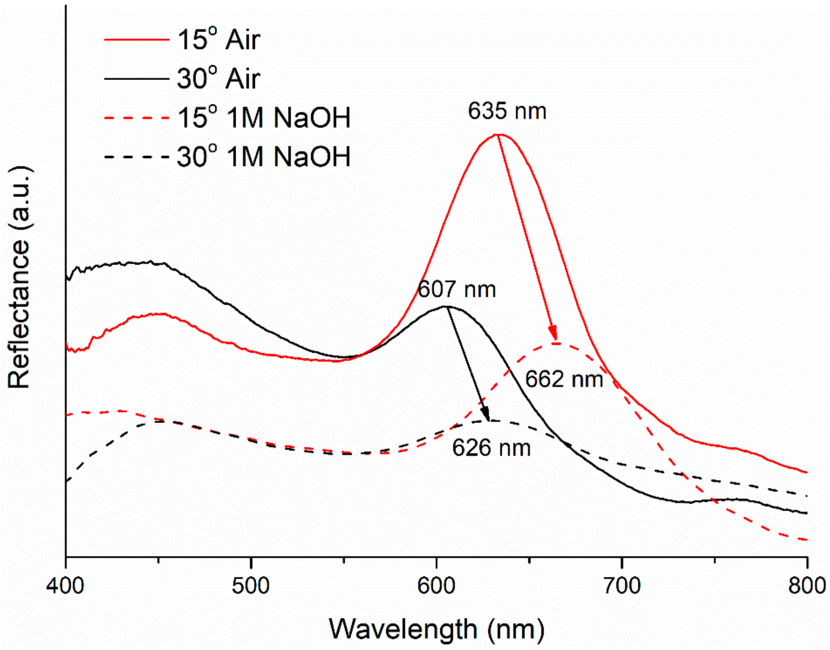

Figure S6. PSB spectra of PC-FTO in NaOH electrolyte.

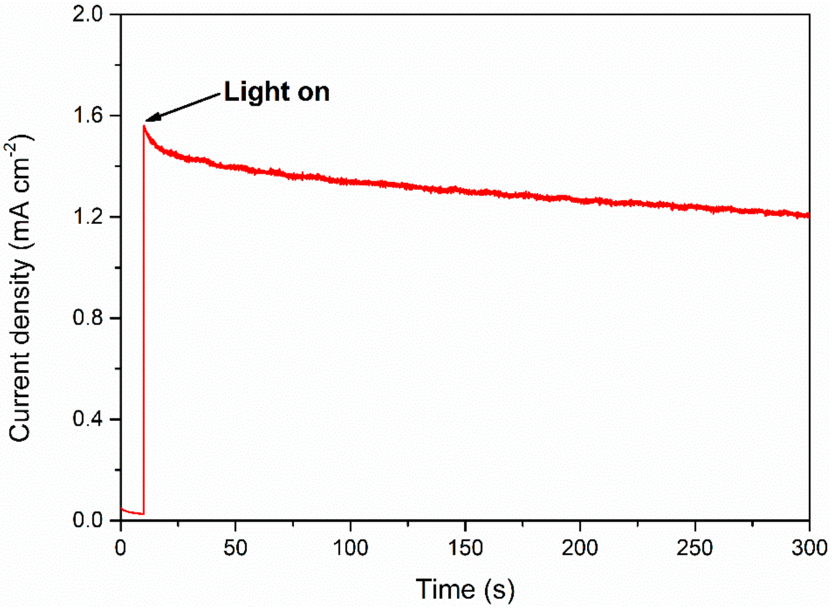

Figure S7. Stability test of Ag_2S/WO_3@PC-FTO electrode under illumination from surface normal for 5 min.

© 2019 by the authors. Submitted for possible open access publication under the terms and conditions of the Creative Commons Attribution (CC BY) license (http://creativecommons.org/licenses/by/4.0/).
